# Life-threatening Chlorhexidine Anaphylaxis: A Case Report

**DOI:** 10.5704/MOJ.1707.008

**Published:** 2017-07

**Authors:** RY Kow, CL Low, JK Ruben, MZ Zaharul-Azri, MS Ng

**Affiliations:** Department of Orthopaedics, Kuala Lipis Hospital, Kuala Lipis, Malaysia

**Keywords:** anaphylaxis, chlorhexidine, life threatening, hypersensitivity

## Abstract

Chlorhexidine is a common antiseptic and disinfectant used in the medical field. Allergy to chlorhexidine has been reported in the literature but life-threatening anaphylactic shock is rare. We present a case of severe anaphylactic shock due to chlorhexidine occurring during surgery. Literatures suggest that profound anaphylactic shock to chlorhexidine is commonly preceded by milder, non-specific reactions. These mild symptoms are often dismissed by both the patient and physicians alike. Direct questioning of these symptoms is necessary as a part of the pre-operative assessment and the patient should be referred for further immunology testing if indicated.

## Introduction

Chlorhexidine is a common antiseptic agent used in the orthopaedic field. Hypersensitivity to chlorhexidine has been reported but life-threatening anaphylactic shock is rare. We present a case of life-threatening anaphylactic shock due to chlorhexidine in a patient occurring during a surgical procedure.

## Case Report

A 20-year old patient with no known medical illness and allergy, sustained an open-book pelvic fracture and closed fractures of the right tibia and fibula following a motor-vehicle accident. He subsequently underwent a surgical fixation of the pelvic, right tibia and fibula fractures under general anaesthesia and the surgery was uneventful. Povidone iodine and chlorhexidine gluconate 0.5% were used as disinfectants intraoperatively. He developed non-infected eczematous rashes at his back and bilateral lower limbs on day one post-operation of which he did not inform to the medical personnel. There was no systemic involvement aside from the cutaneous manifestation. He was discharged home on post-operative day five and the rashes subsided at home without any sequela.

Two months later, he had another surgery under general anaesthesia for debridement of the right leg infected wound and pelvic external fixator removal. Povidone iodine and chlorhexidine gluconate 0.5% were used as disinfectants of the wounds intraoperatively. Towards the end of the surgery, he developed severe bronchospasm followed by pulseless electrical activity (PEA). He had facial flushing, angioedema of the lips and flushing of bilateral upper and lower limbs with periorbital, hands, and feet oedema. Cardiac resuscitation was commenced immediately. There was recovery of circulation after five minutes of PEA and a total of 5mg intravenous (IV) adrenaline was given. IV hydrocortisone and chlorpheniramine were given as part of the immediate management. Post-operatively, he was treated at the intensive care unit (ICU) with triple inotropes. He subsequently recovered and was discharged home well.

He had negative intradermal testing to validated standard concentrations of fentanyl, propofol, morphine, suxamethonium, rocuronium, atracurium, chlorhexidine and povidone iodine. Skin prick tests with latex, fentanyl, propofol and muscle relaxants were all negative. However, his specific Ig E to chlorhexidine was found to be raised to 0.77 kUA/L (reference <0.35kUA/L). Morphine-based immunoassay which is used to detect sensitization against the antigenic quaternary ammonium (NH4^+^) epitope of neuromuscular blocking agents was negative. Latex specific Ig E (rHev B8 and rHev B6) is within normal range. A repeat intradermal testing with validated strong concentrations of fentanyl, propofol, morphine, suxamethonium, rocuronium, atracurium, chlorhexidine and povidone iodine two months later revealed a wheal with flare at the chlorhexidine intradermal site. A repeat skin prick test to chlorhexidine was also positive (Table I).

**Table I:** Skin prick test and intradermal test, and specific Ig E results

**Reagent**	**Skin prick test**	**Intradermal test (standard concentration)**	**Specific Ig E (Reference <0.35KUA/L)**	**Skin prick test (repeated)**	**Intradermal test (strong concentration)**
Neuromuscular Blocking Agents					
Atracurium	Negative	Negative	Not available	Negative	Negative
Suxamethonium	Negative	Negative	Not available	Negative	Negative
Rocuronium	Negative	Negative	Not available	Negative	Negative
Hypnotics					
Propofol	Negative	Negative	Not available	Negative	Negative
Analgesic Agents					
Fentanyl	Negative	Negative	Not available	Negative	Negative
Morphine**	Negative	Negative	Negative	Negative	Negative
Antiseptic Agents					
Povidone-iodine	Negative	Negative	Not available	Negative	Negative
Chlorhexidine	Negative	Negative	0.77 KUA/L	Positive	Positive
Other					
Latex*	Negative	-	rHev B8 negative		
			rHev B6 negative	Negative	-

^*^ Recombinant latex allergens are used to test for latex-specific Ig E level.

^**^ Morphine-based immunoassay is used to detect sensitization against the antigenic quaternary ammonium (NH4_+_) epitope of neuromuscular blocking agents.

^***^ Antibiotics are not tested as the patient is given regular doses of cefuroxime in the ward without any complication.

## Discussion

Chlorhexidine is a commonly used antiseptic and disinfectant in the medical field^1^. In orthopaedics, it is commonly used both pre-operatively and post-operatively. It is a cationic bisguanide, used in the form of (di) acetate or (di) gluconate salts^2^. Chlorhexidine salts may trigger local or systemic allergic reactions. Allergy to chlorhexidine has been reported in the literature, but life-threatening anaphylactic shock is rare^3^. Life-threatening anaphylaxis is commonly associated with mucosal and parenteral exposure^2^. It is a type 1 hypersensitivity reaction, which is associated with the synthesis of Ig E antibodies^1^. When a patient is exposed to a triggering chlorhexidine antigen, Ig E antibodies are produced from B lymphocytes. The Ig E produced then bind to the surface membranes of mast cells for “priming” effect^1^. When the patient is exposed to chlorhexidine antigens for a second time, the antigens bind rapidly to the Ig E antibodies of primed mast cells, causing the mast cells to degranulate and release immunologic mediators such as histamine, cytokines, and leukotrines^1^.

Adverse reactions to chlorhexidine range from contact dermatitis and generalized urticaria to life-threatening anaphylactic shock. Literatures suggest that profound anaphylactic shock to chlorhexidine is commonly preceded by milder, non-specific reactions^4^. These mild symptoms are often dismissed by both the patient and physicians alike. Direct questioning of these symptoms is necessary as a part of the pre-operative assessment. Resuscitation drugs such as adrenaline and hydrocortisone are needed and have been reported in cases with successful treatment^3^. Therefore, in the case of an anaphylaxis occurring during an emergency surgery, immediate treatment with intravenous administration of hydrocortisone and antihistamine as well as intramuscular adrenaline injection must be initiated. In our patient, he was given intravenous adrenaline in view of pulseless electrical activity (PEA) occurring during the anaphylactic shock. Endotracheal intubation for airway protection with ventilatory and haemodynamic supports associated with invasive monitoring in an intensive care unit post-operatively are needed in cases of anaphylactic shock.

In our patient, he was sensitized to the chlorhexidine antigen during the first surgery, and this explained the rashes developed after the first surgery. He suffered from an anaphylactic shock during the second surgery when he was again exposed to the same antigen. Upon discharge, the patient was referred to a tertiary center for immunology testing to look for the causative antigens and all investigations were negative except for chlorhexidine.

Ig E-mediated reactions to chlorhexidine are uncommon among patients with suspected contact dermatitis. The incidence ranges from 0.47% to 5.2% in the literature^5^. However, it is important to perform the immunology testing to exclude other more common causes of Ig E-mediated reactions in suspected patients to prevent anaphylaxis. The most common cause of Ig E-mediated reaction is neuromuscular blocker agents (more than 60%), drugs commonly used during general anaesthesia^5^. However, the causative agent(s) may be difficult to be determined during an anaphylactic emergency, hence a proper immunology testing that includes all the suspected agents needs to be carried out after the acute event to prevent another episode of life-threatening anaphylaxis. In an emergency setting, an immunology testing is usually not possible to be performed.

Hence, in those patients with a history of severe anaphylactic reactions to unknown drugs, it is important to weigh the risk of another life-threatening anaphylactic shock with the benefits of the surgery. Until a causative agent is identified, a properly planned surgery can reduce the risks of the surgery.

## Conclusion

Life-threatening chlorhexidine anaphylaxis is rare but it is normally preceded by milder symptoms. Direct questioning of these symptoms is necessary as a part of the pre-operative assessment and the patient should be referred for further immunology testing if indicated.
